# Supplementary material for: Design and Synthesis of Benzene Homologues Tethered with 1,2,4-Triazole and 1,3,4-Thiadiazole Motifs Revealing Dual MCF-7/HepG2 Cytotoxic Activity with Prominent Selectivity via Histone Demethylase LSD1 Inhibitory Effect
Source: Int J Mol Sci. 2022 Aug 8;23(15):8796. doi: 10.3390/ijms23158796 (PMC9369007; doi:10.3390/ijms23158796)
Supplement: Supplementary file 1 [file ijms-23-08796-s001.zip › ijms-1800360-supplementary.pdf]

Supplementary materials

Spectroscopic data

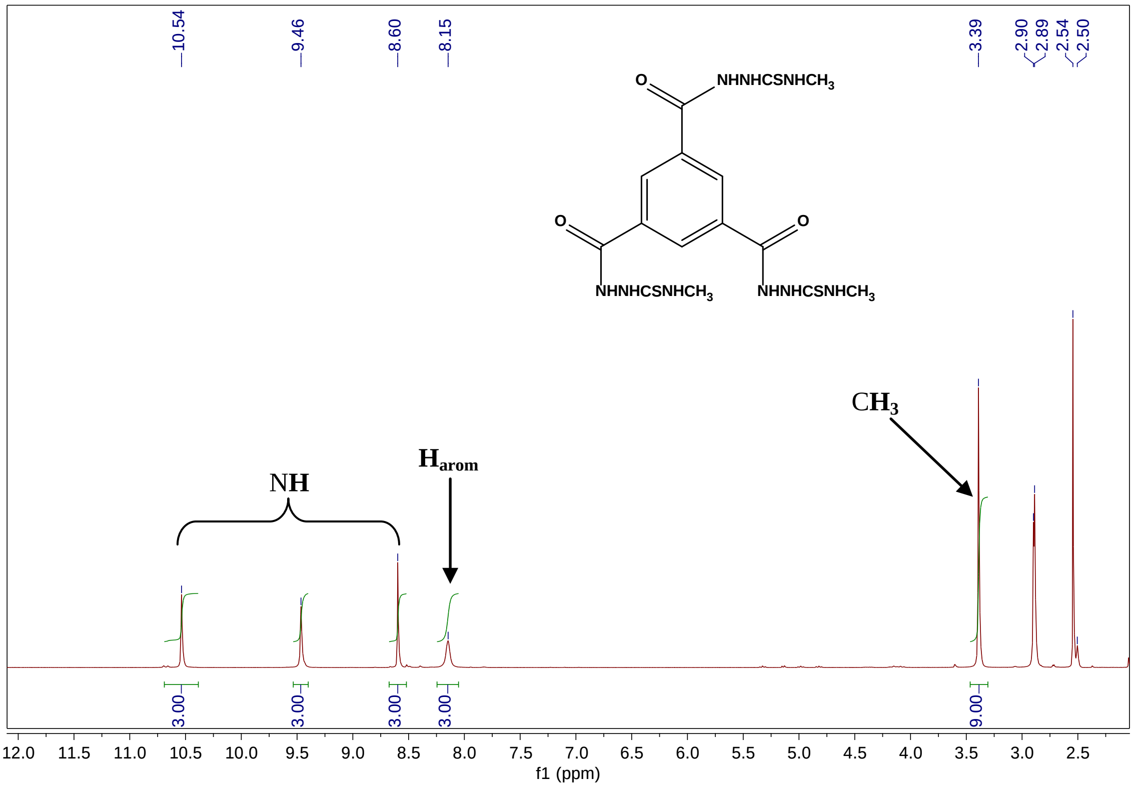

Figure S1: ^{1}H NMR spectrum of compound **12**

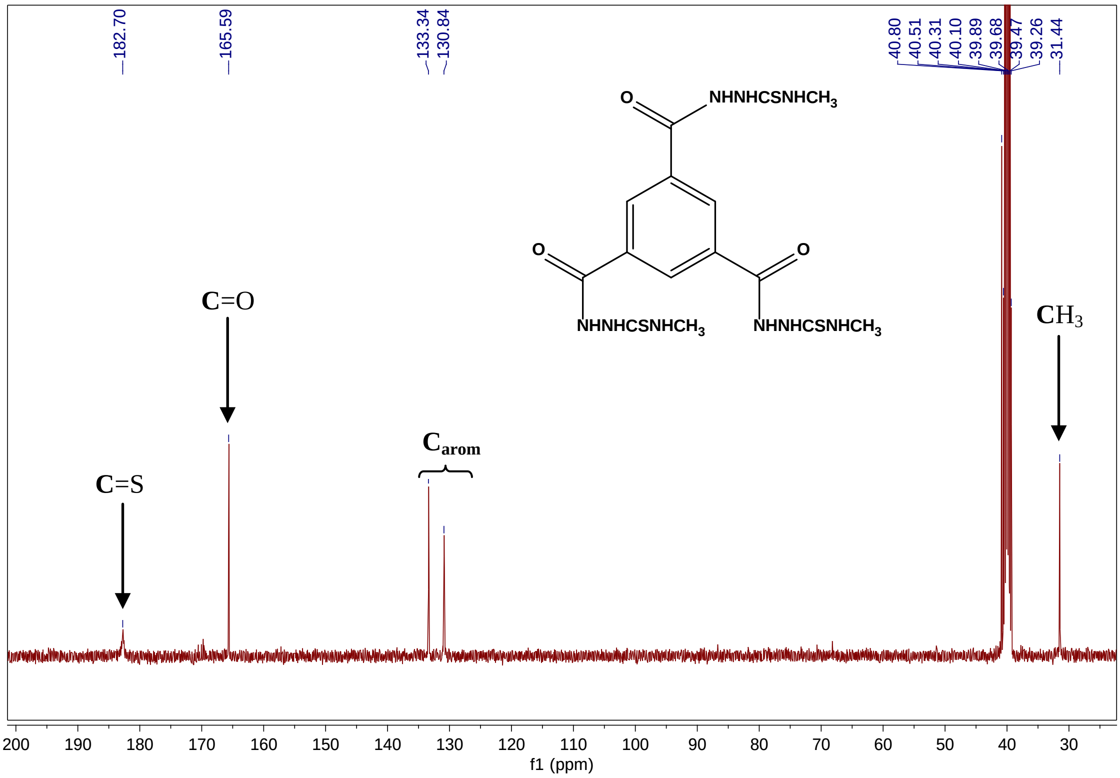

Figure S2: ^{13}C NMR spectrum of compound **12**

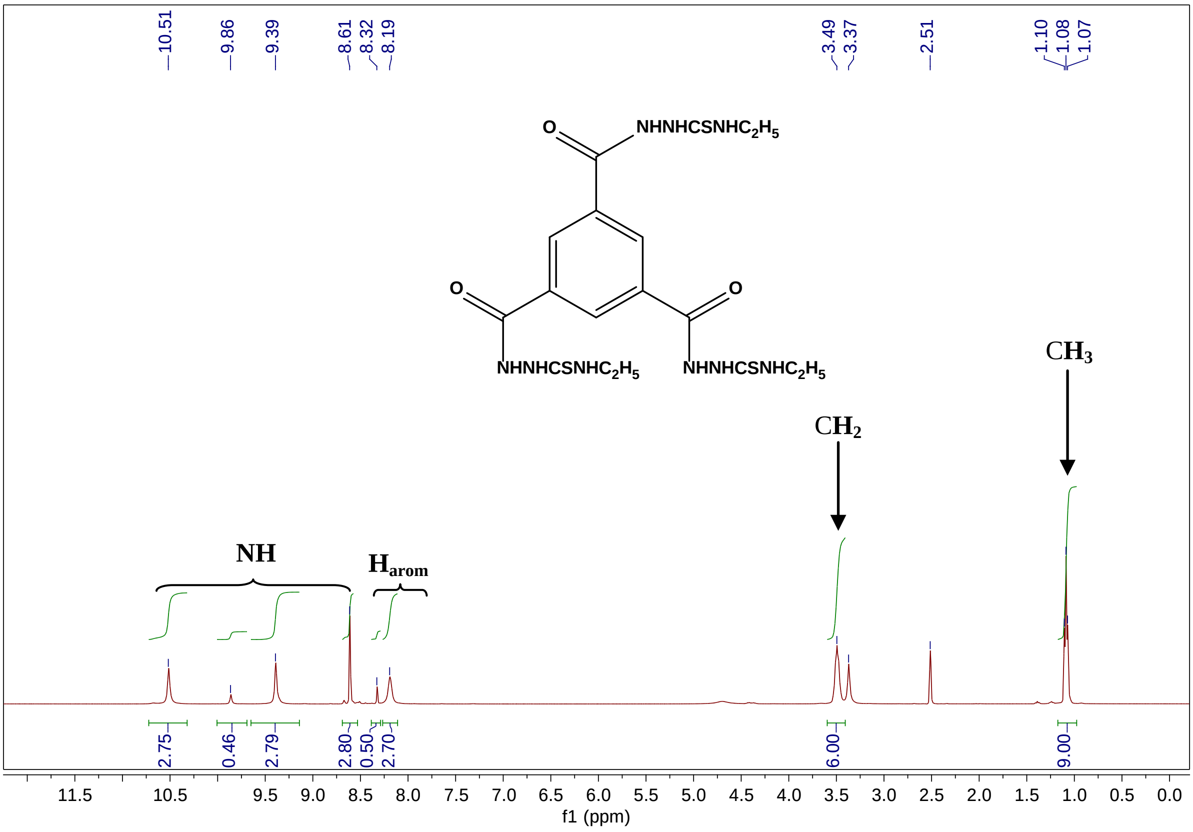

Figure S3: ^{1}H NMR spectrum of compound **13**

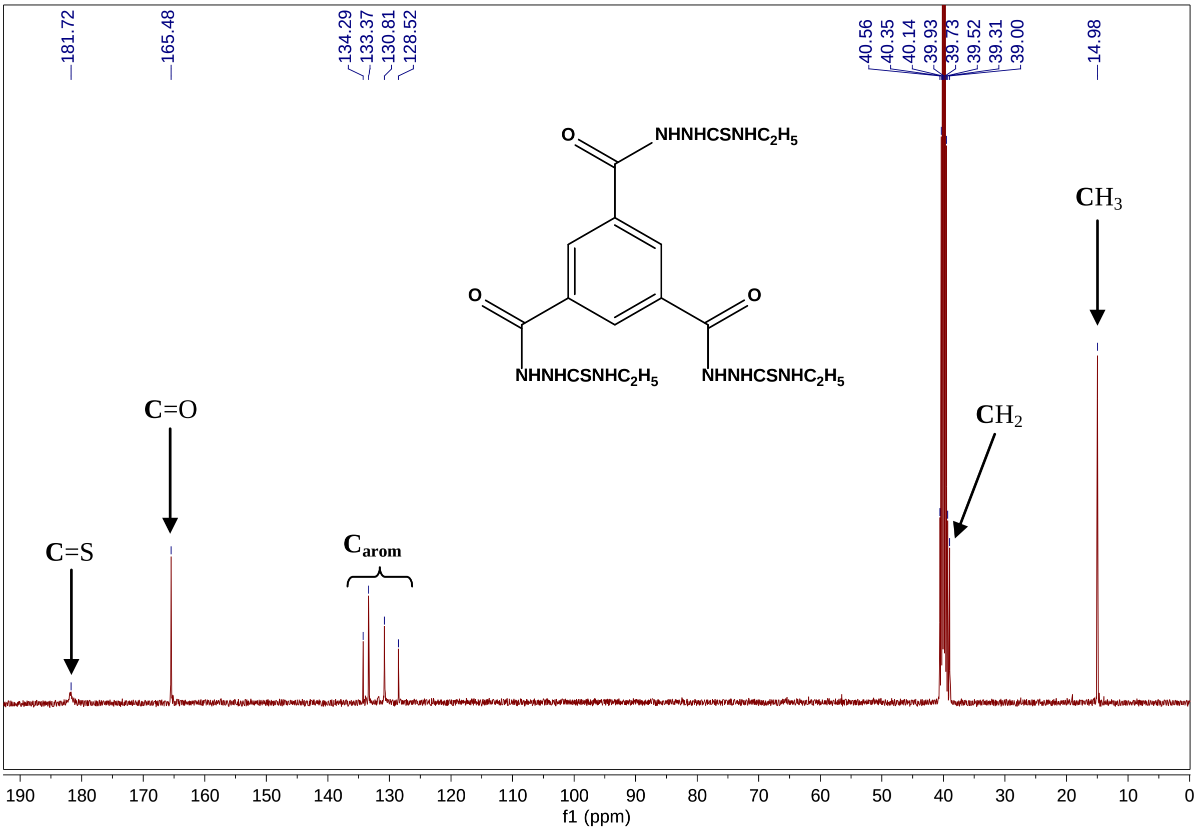

Figure S4: ^{13}C NMR spectrum of compound **13**

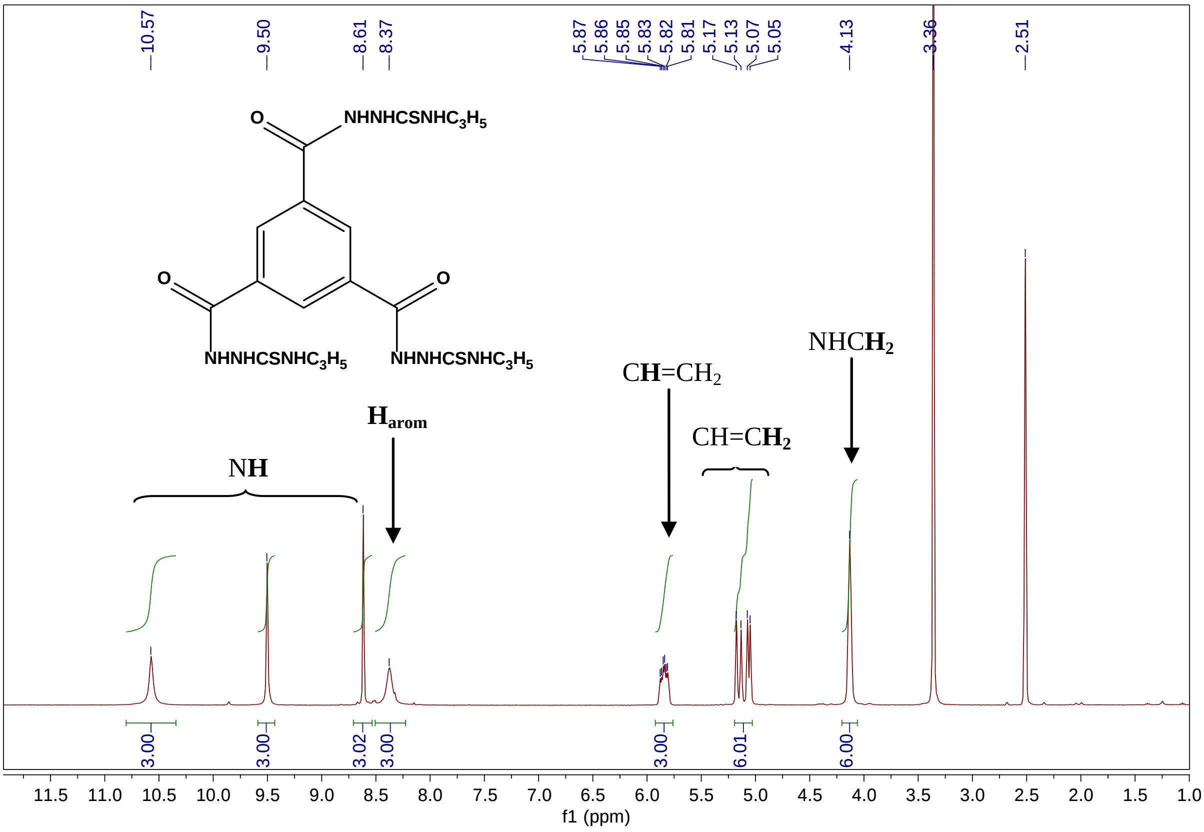

Figure S5: ^{1}H NMR spectrum of compound **15**

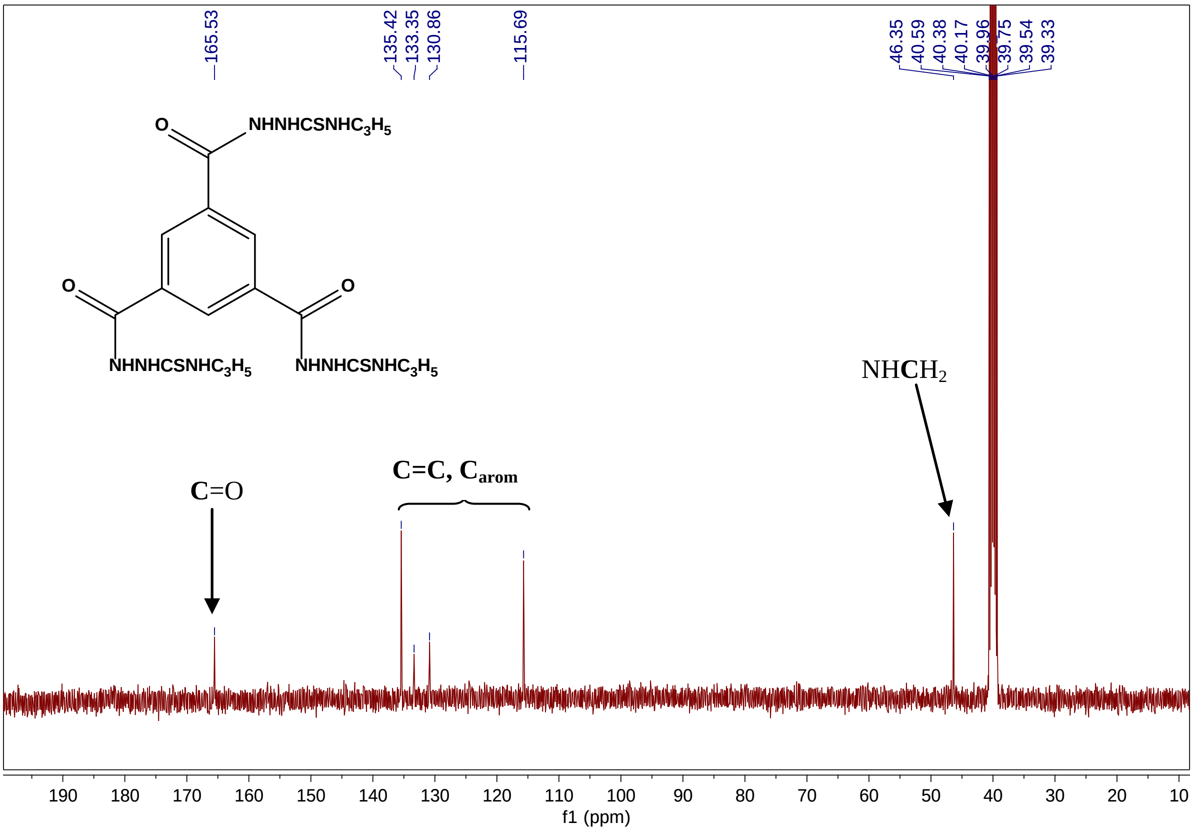

Figure S6: ^{13}C NMR spectrum of compound **15**

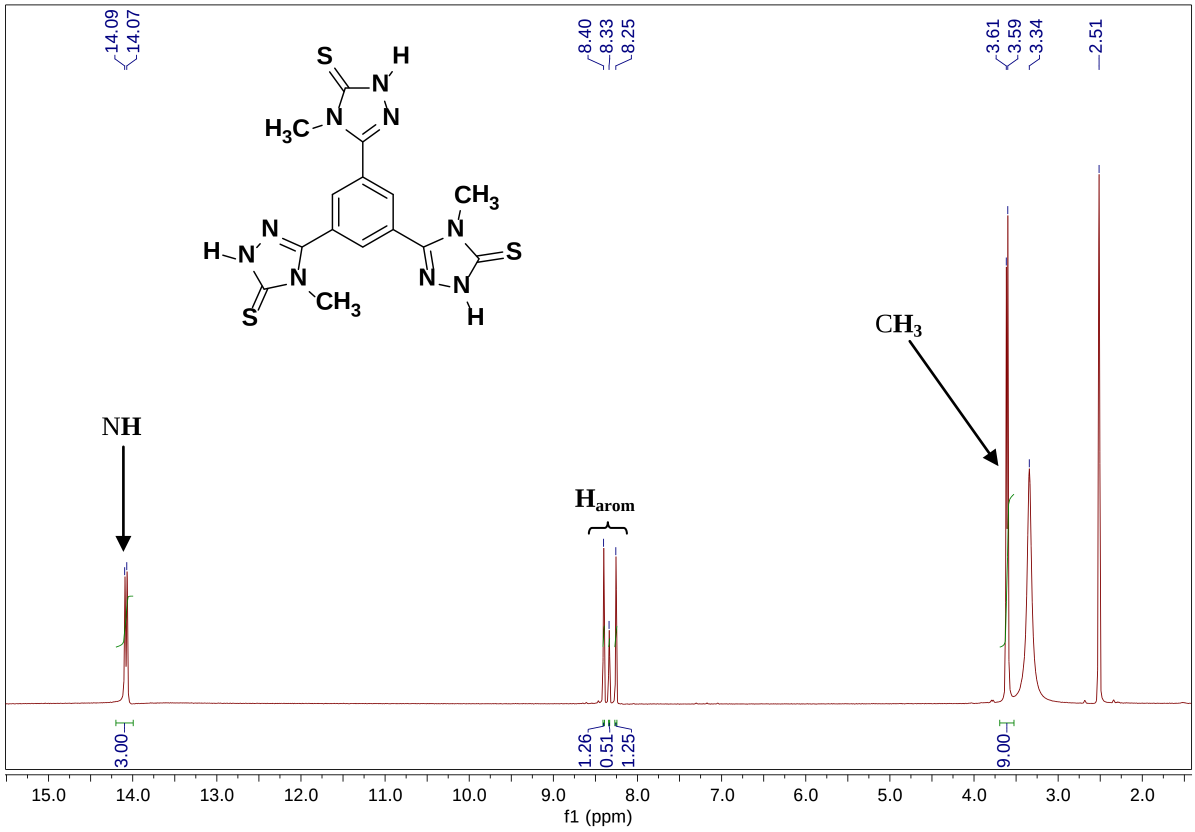

Figure S7: ^{1}H NMR spectrum of compound **18**

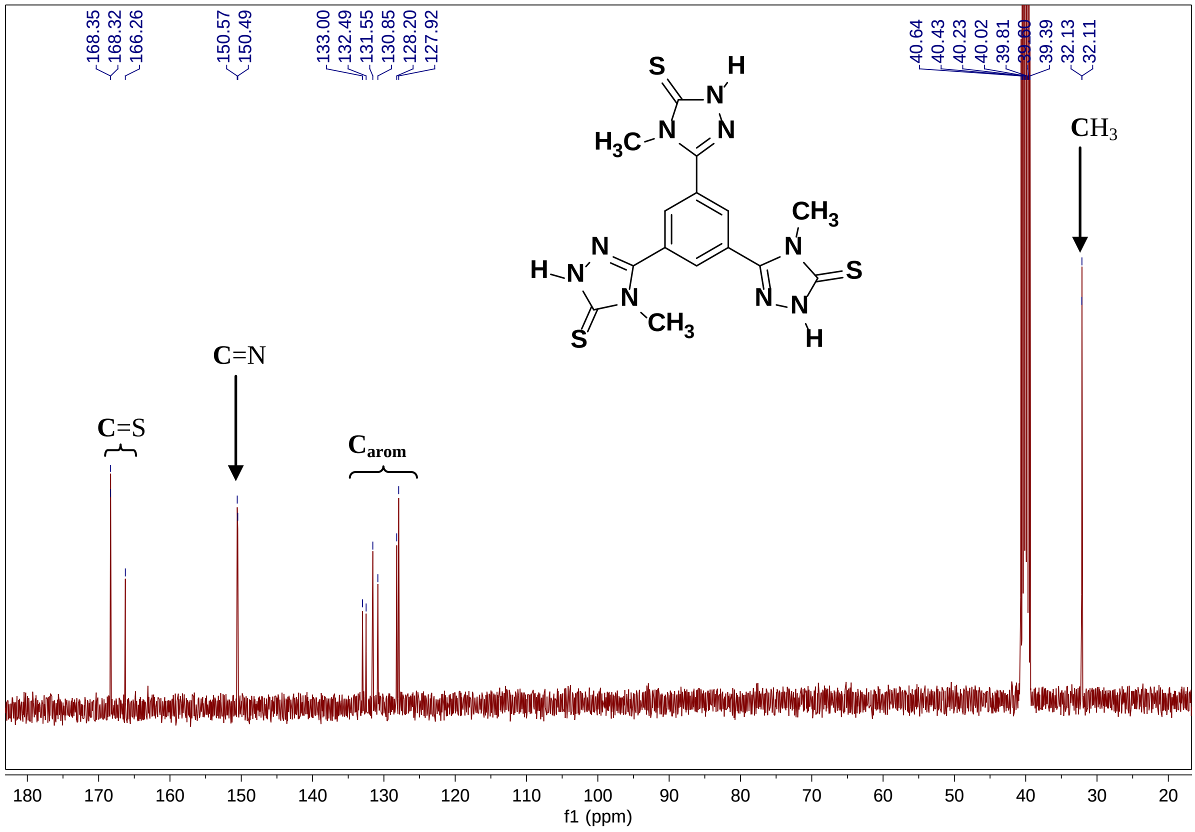

Figure S8: ^{13}C NMR spectrum of compound **18**

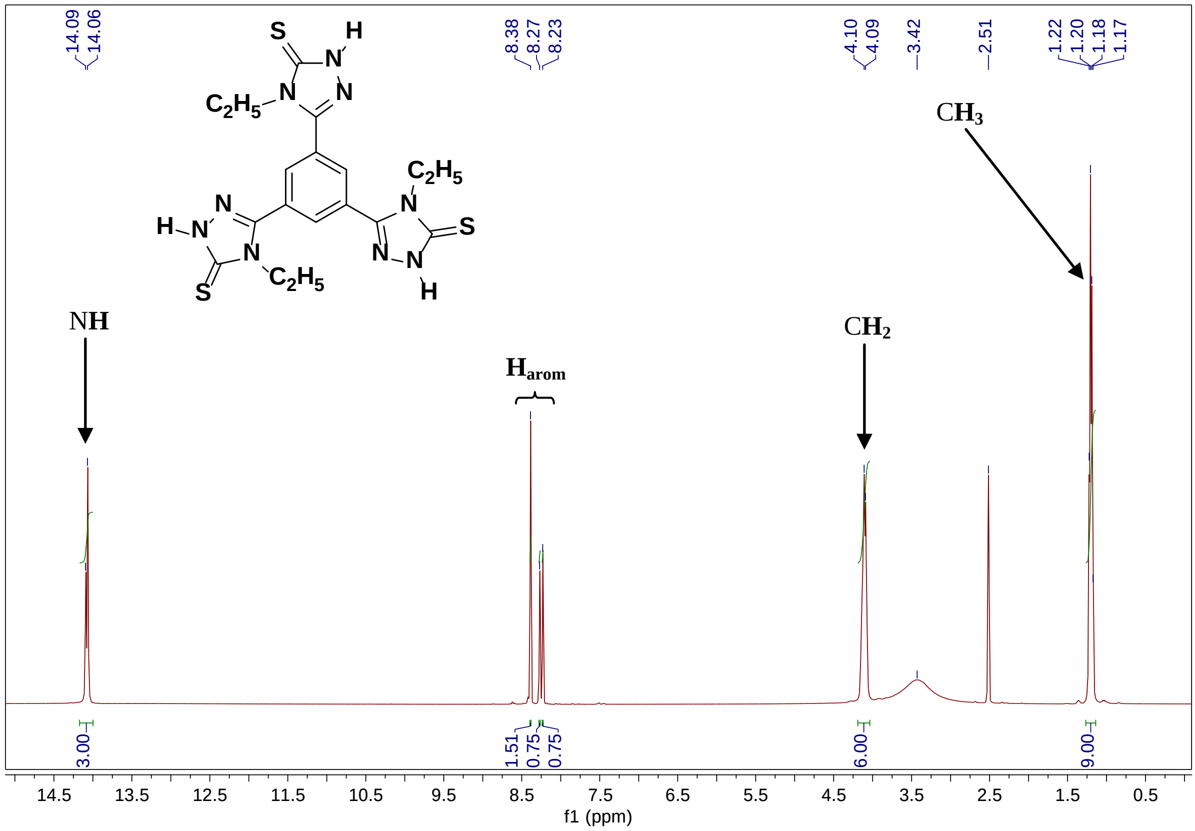

Figure S9: ^{1}H NMR spectrum of compound **19**

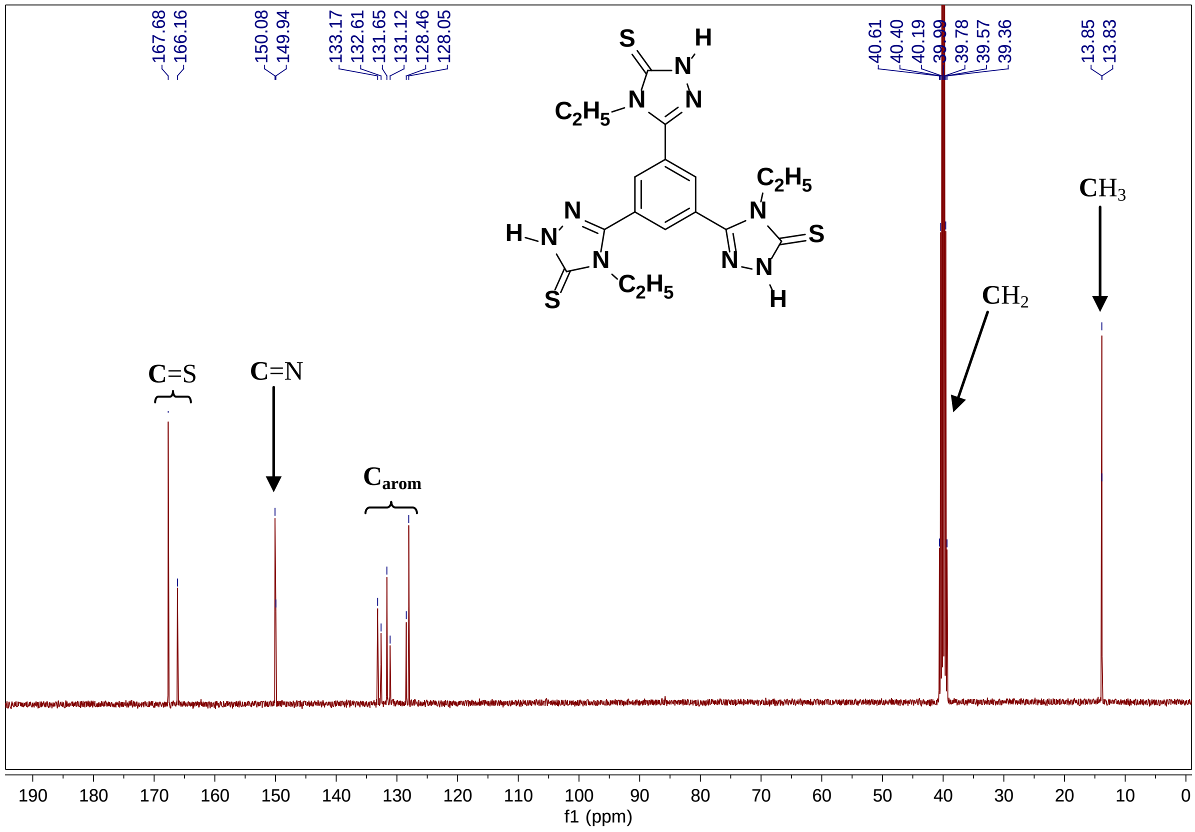

Figure S10: ^{13}C NMR spectrum of compound **19**

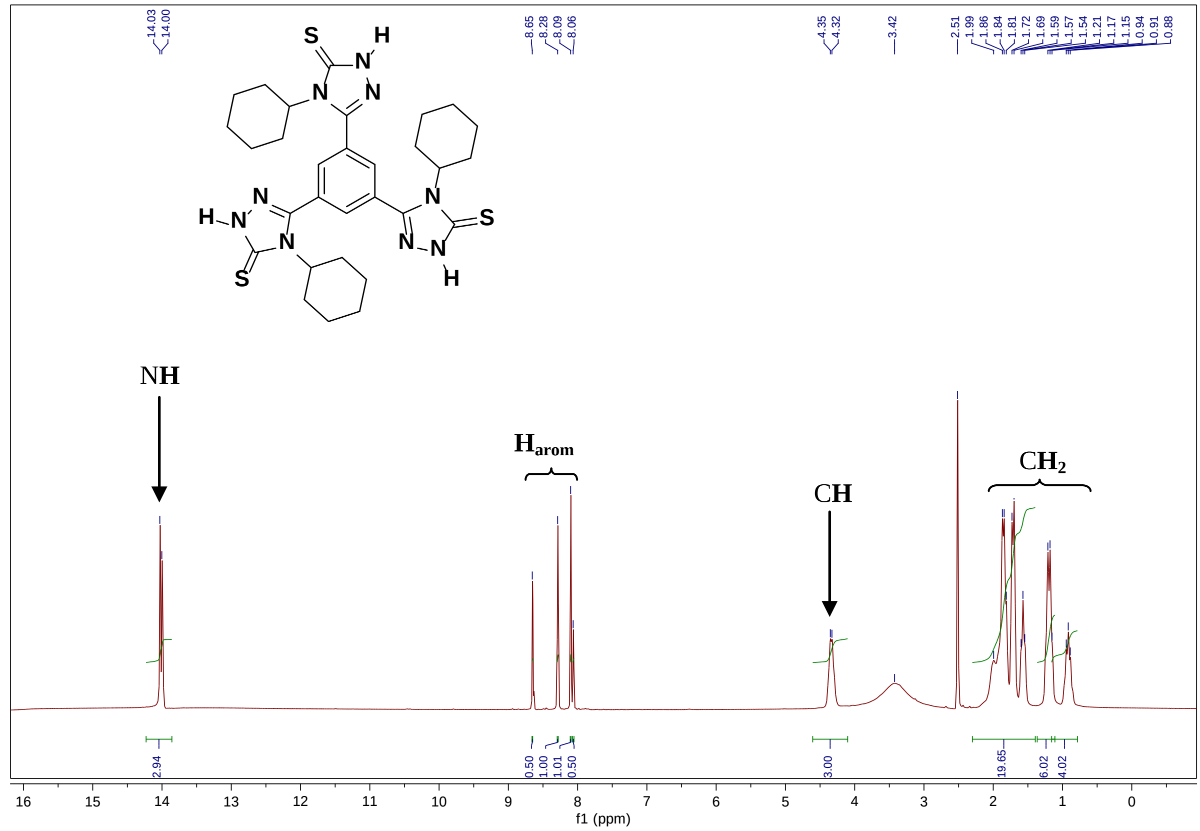

Figure S11: ^{1}H NMR spectrum of compound **20**

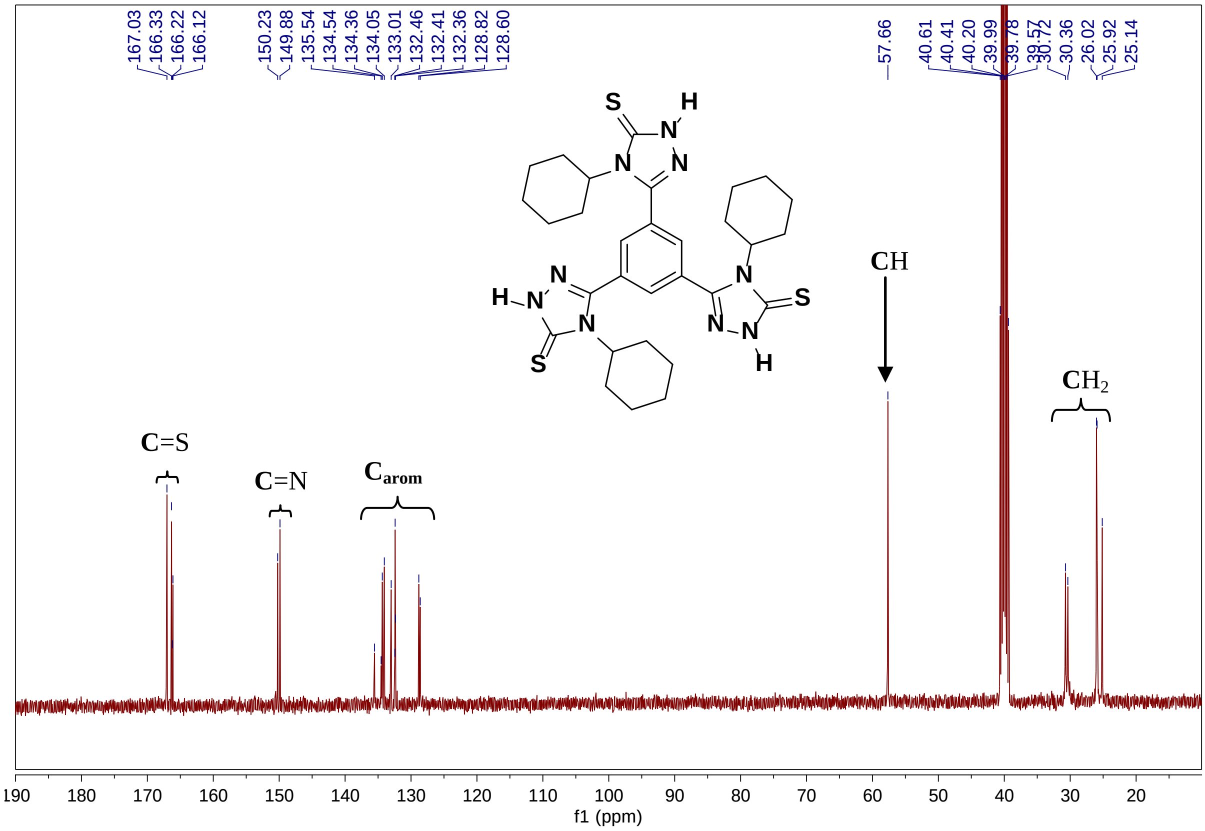

Figure S12: ^{13}C NMR spectrum of compound **20**

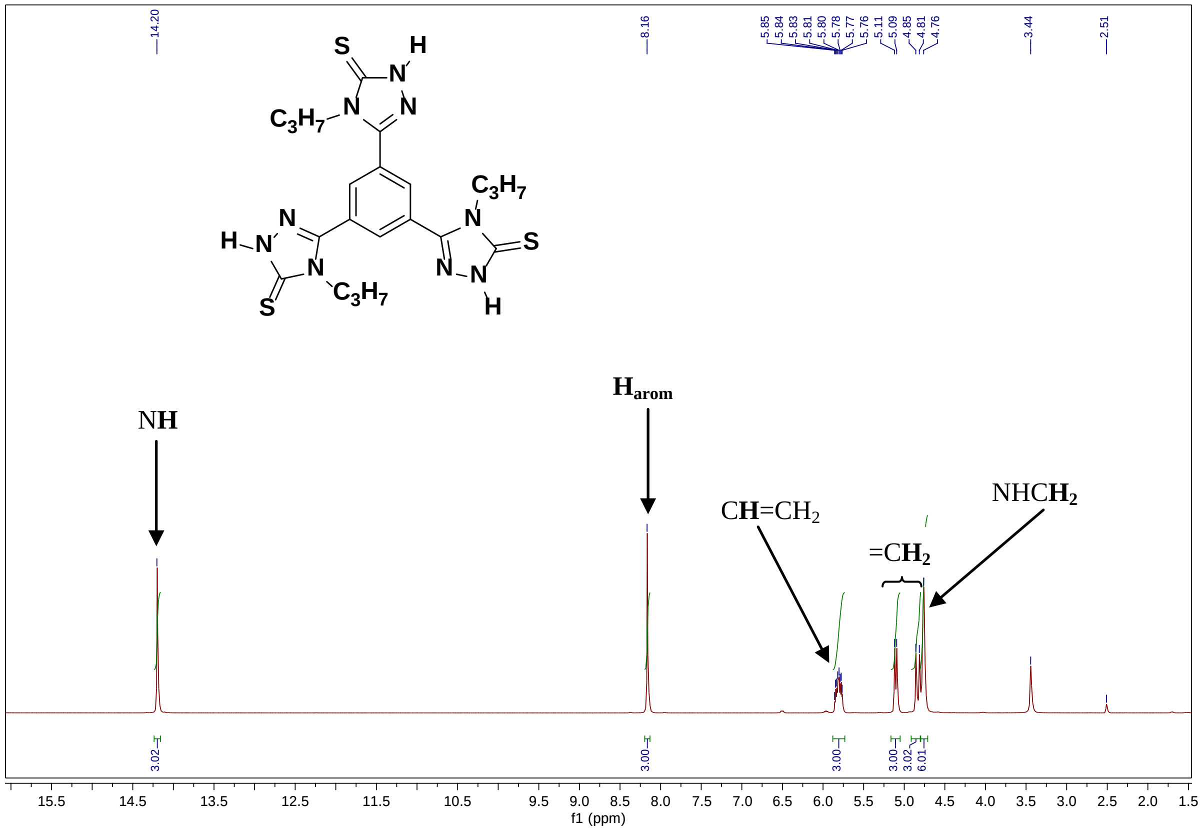

Figure S13: ^{1}H NMR spectrum of compound **21**

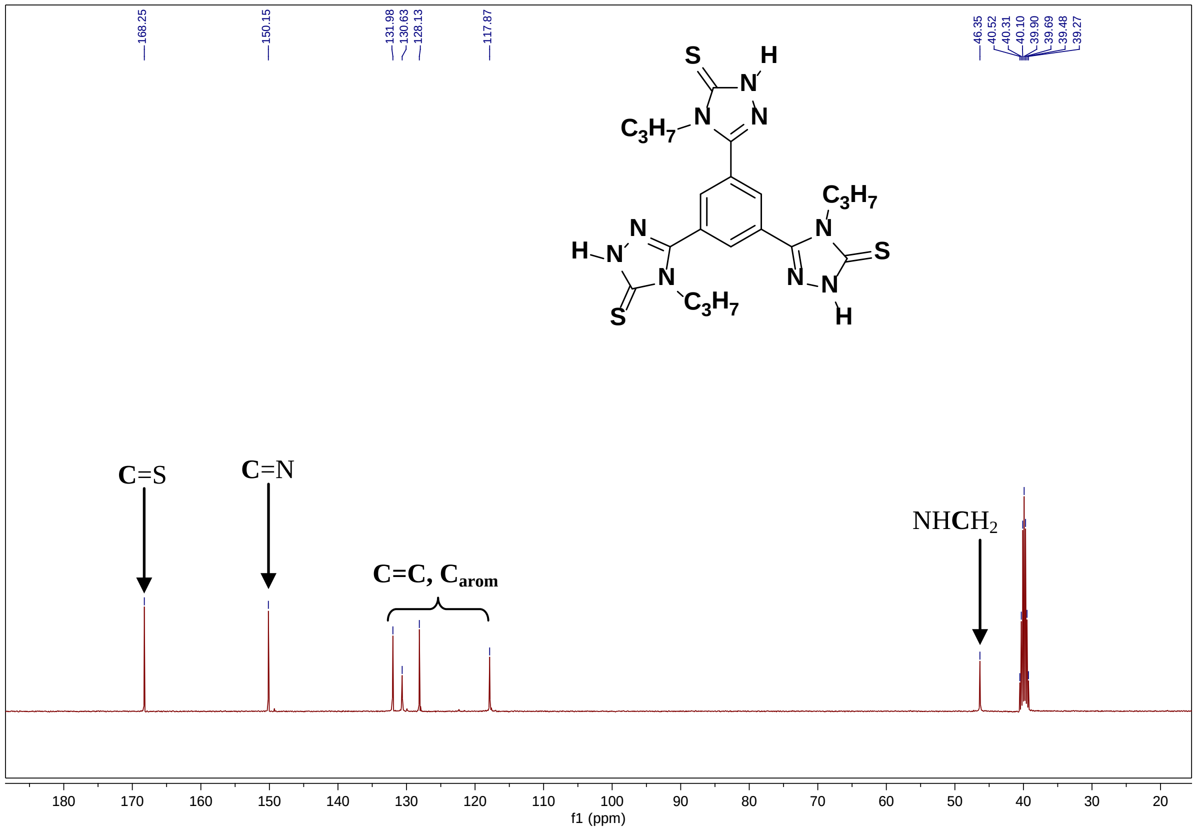

Figure S14: ^{13}C NMR spectrum of compound **21**

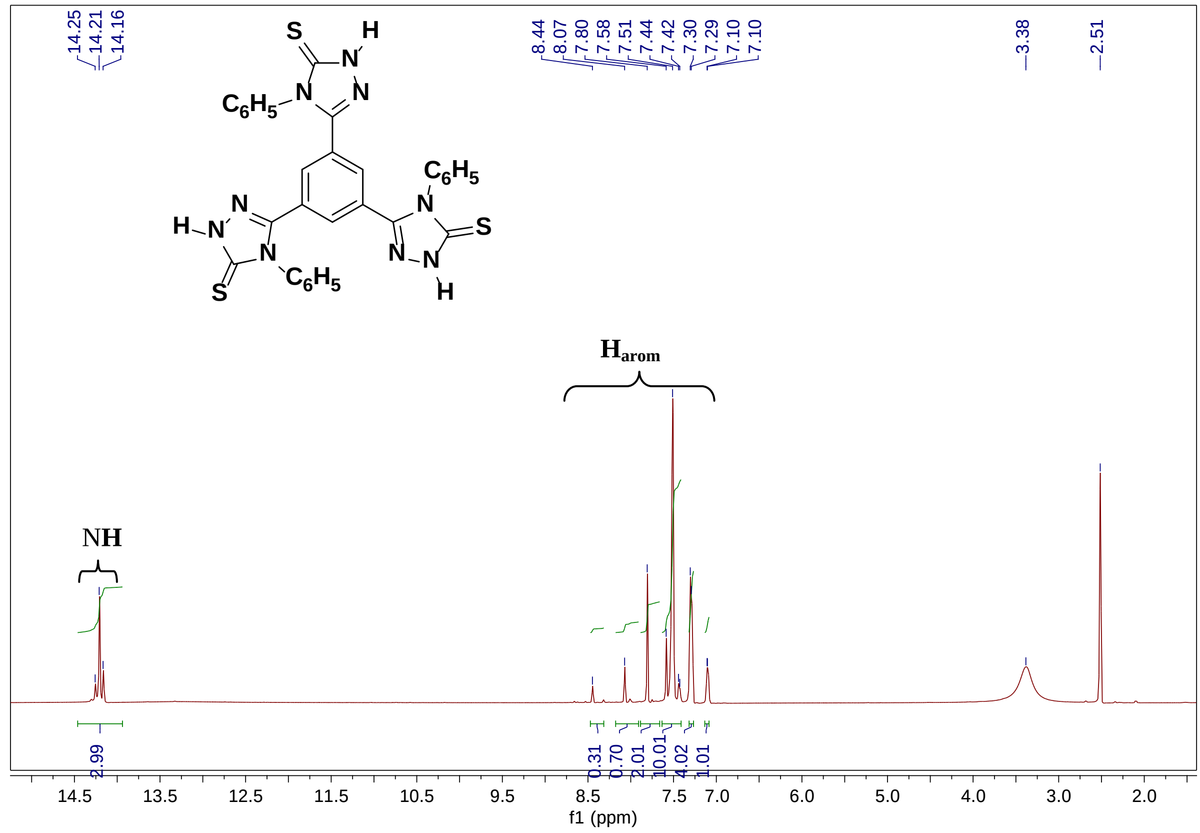

Figure S15: ^{1}H NMR spectrum of compound **23**

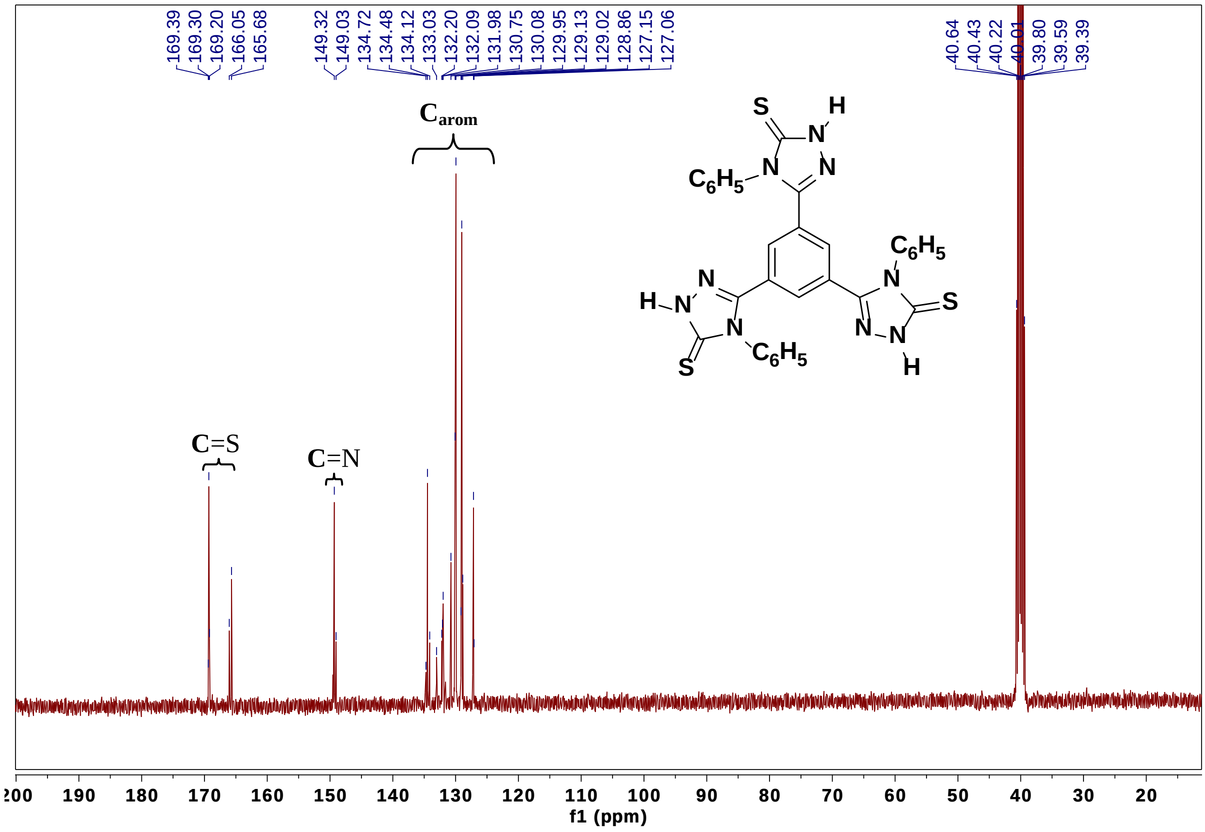

Figure S16: ^{13}C NMR spectrum of compound **23**

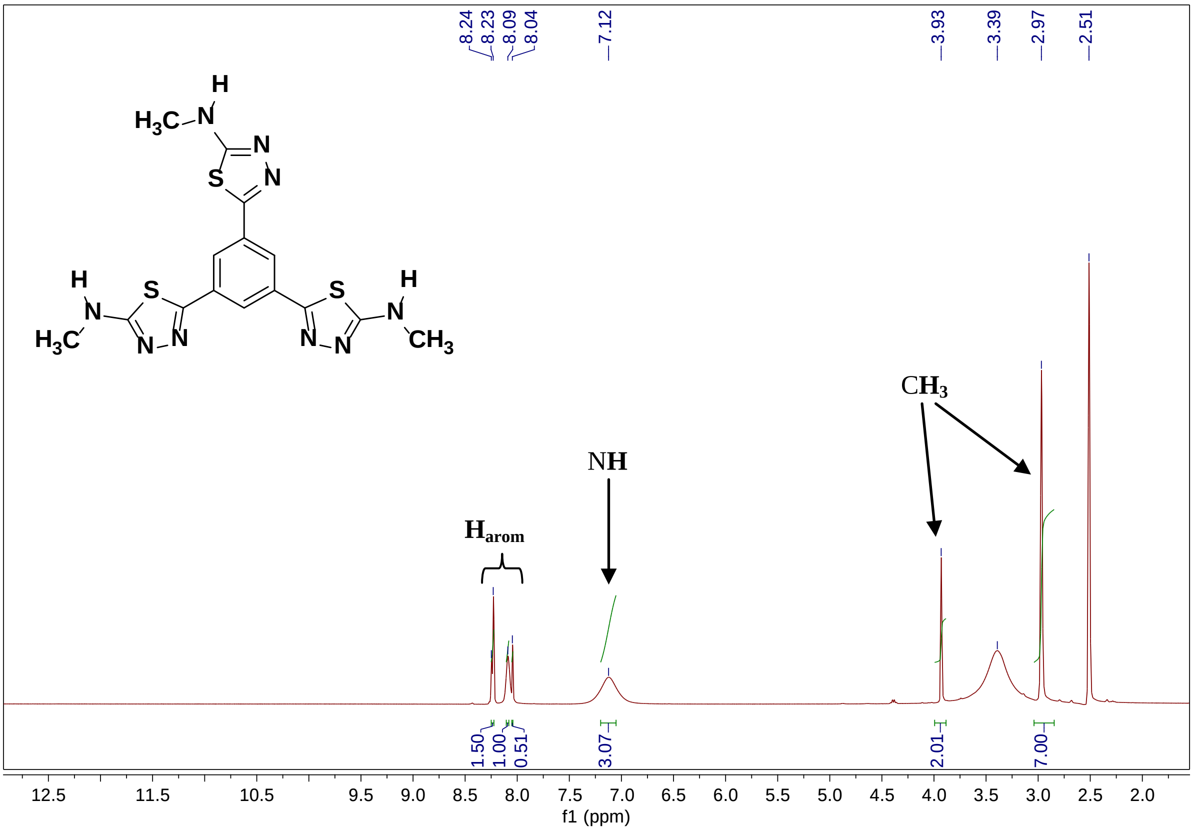

Figure S17: ^{1}H NMR spectrum of compound **24**

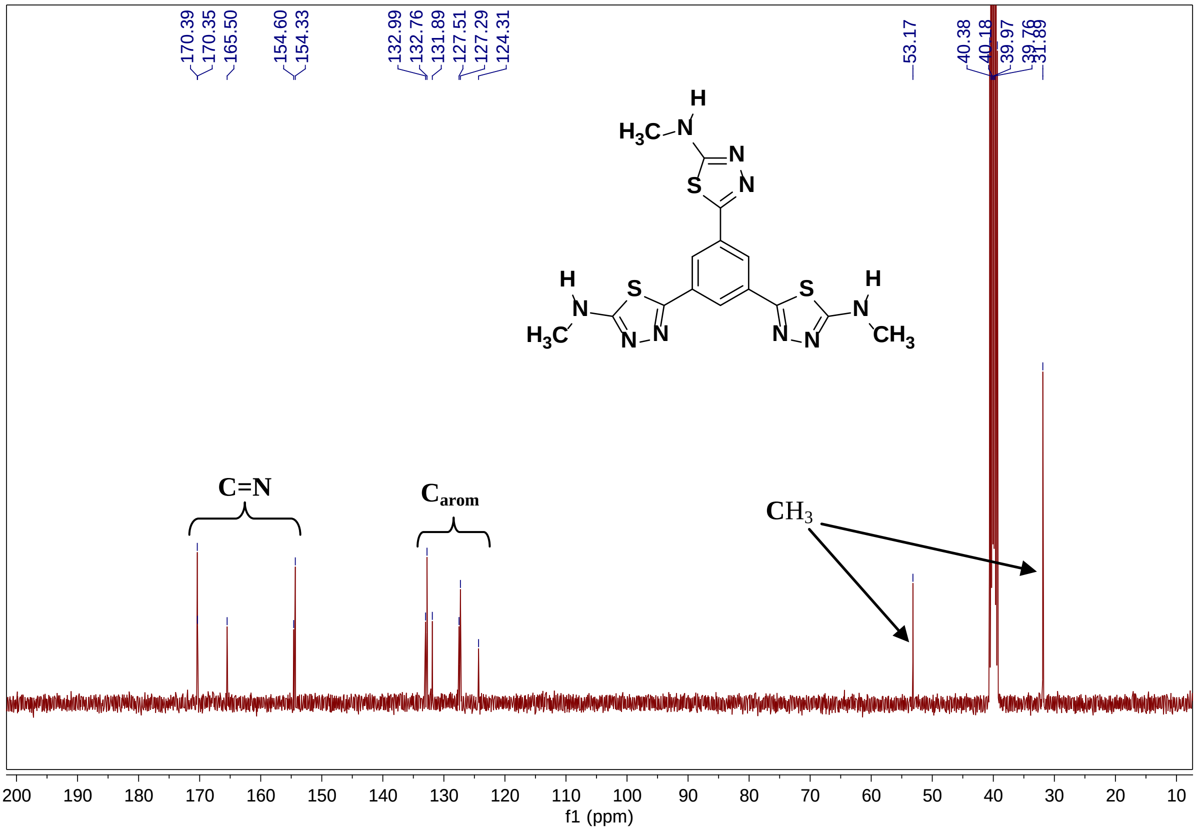

Figure S18: ^{13}C NMR spectrum of compound **24**

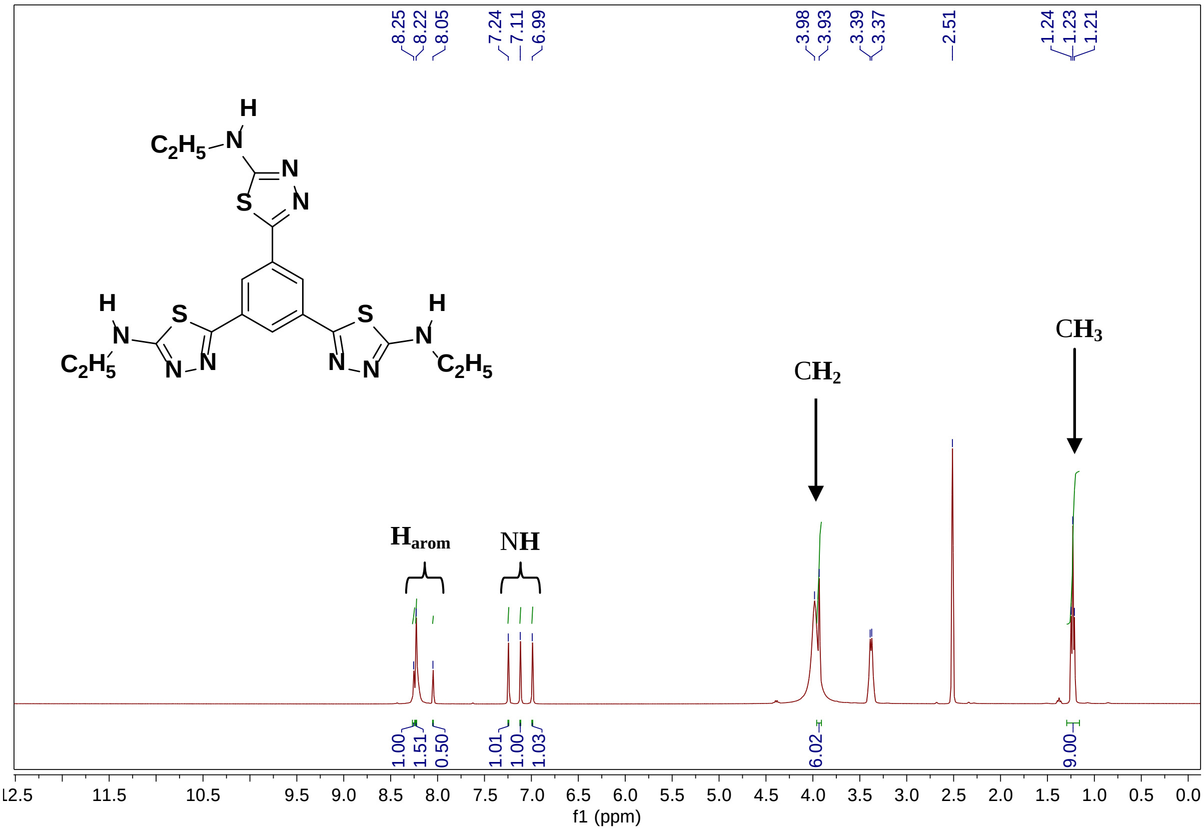

Figure S19: ^{1}H NMR spectrum of compound **25**

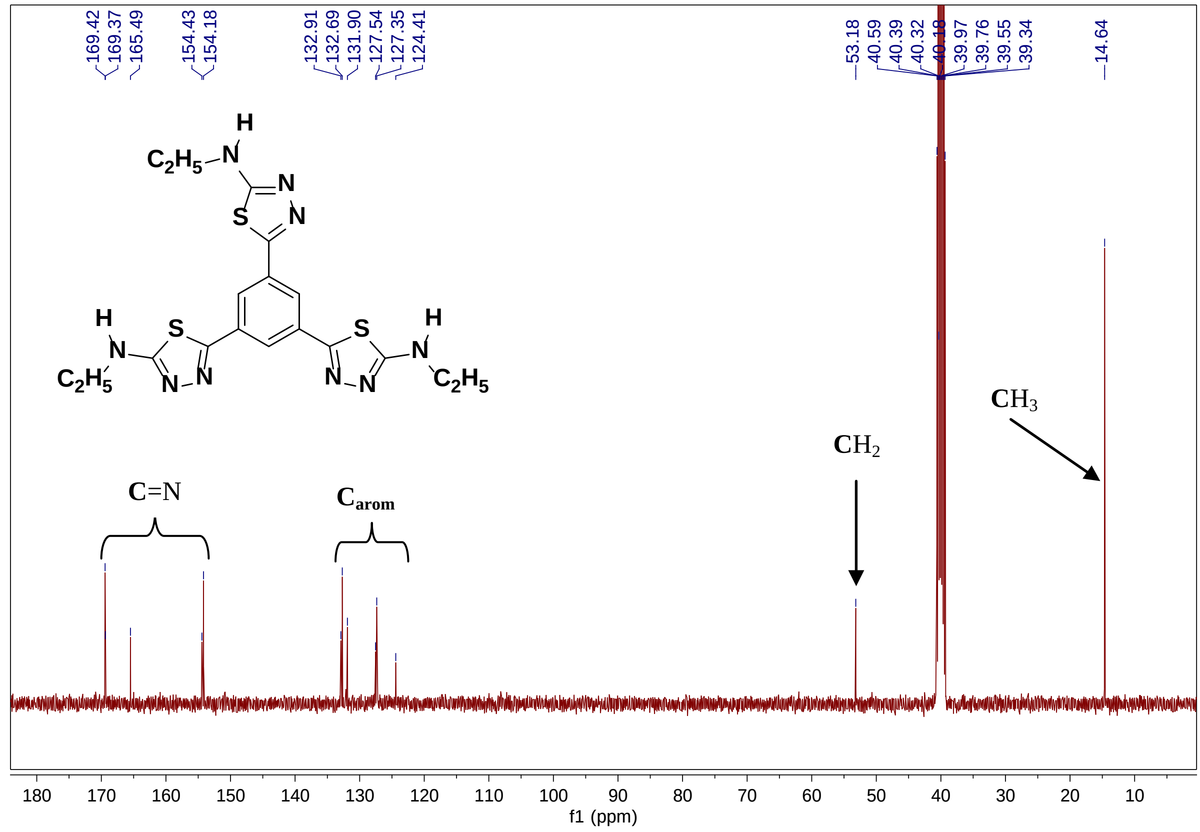

Figure S20: ^{13}C NMR spectrum of compound **25**
